# Minimizing the risk of *Clostridioides difficile* infection as an early complication of autologous stem cell transplantation

**DOI:** 10.1017/ash.2023.424

**Published:** 2023-09-18

**Authors:** Joseph Van Galen, Samuel Maldonado, Kyle Grose, Francis Bagley, Rachele Olivier, Jenna Van Hoose, Michael Keng, Leonid Volodin

**Affiliations:** 1 Internal Medicine Residency Program, University of Virginia, Charlottesville, VA, USA; 2 Pharmacy Services, University of Kansas Health System, Kansas City, KS, USA; 3 Division of Hematology and Oncology, University of Virginia, Charlottesville, VA, USA

**Keywords:** *Clostridium difficile*, stem cell transplantation, quality improvement, patient safety

## Abstract

This quality improvement project aimed to reduce institutional incidence of *Clostridioides difficile* infection (CDI) following autologous stem cell transplantation. CDI incidence per transplant was .17 in a baseline period and .09 following the implementation of postdischarge ultraviolet room cleaning (χ^2^ = 2.11, *p* = .15).

## Introduction

American academic institutions have reported incidences of *Clostridioides difficile* infection (CDI) after autologous stem cell transplantation (SCT) ranging from .065 to .09 per transplant, with most cases occurring in the first month after transplantation.^1–4^ Although CDI incidence is higher following allogeneic transplant, median onset of disease is sooner following autologous stem cell transplant (6.5 vs. 33 days posttransplant).^1,5^ Mortality associated with CDI is higher among recipients of autologous stem cell transplants than among other hospitalized patients, though comparisons of complication rates between recipients of autologous and allogenic stem cell transplants are inconclusive.^5,6^ This study aimed to reduce CDI incidence by one-third and to reduce length of stay (LOS) after autologous stem cell transplant.

## Methods

Data were collected from patients who underwent autologous SCT at the University of Virginia Medical Center between June 2016 and July 2019. Standard practice prior to this study was administration of ciprofloxacin, 500 mg twice daily unless contraindicated, from transplantation until engraftment (absolute neutrophil count >500). A multidisciplinary team composed of Oncology and Infectious Diseases physicians, pharmacists, nurses, and Environmental Services professionals used quality improvement tools including process mapping and priority matrices to identify feasible interventions. Data were first collected for a baseline period from the start of the study until the start of the first plan-do-study-act (PDSA) cycle (June 2016–August 2017). In a first PDSA cycle (August 2017–May 2018), routine use of prophylactic ciprofloxacin was suspended after the intervention had been reviewed and waived by the University of Virginia Institutional Review Board for Health Sciences Research. In a second PDSA cycle (June 2018–July 2019), ultraviolet (UV) cleaning tools were added to the postdischarge cleaning protocol, using the Surfacide Helios UV System with room-sensing feature for run time determination. Chemical cleaning included sporicidal agents throughout both PDSA cycles. Ciprofloxacin was reimplemented soon after the start of PDSA#2, so as to isolate the effect of one intervention at a time, relative to our baseline period. *Clostridioides difficile* testing was performed at the discretion of faculty and housestaff using Cephid platform PCR testing for *tcdB* upon development of symptoms. CDI incidence and posttransplantation LOS were retrospectively audited. Statistical process control (SPC) analyses were performed using QIMacros 2021.07 with 3σ control limits after grouping patients into sixty-day cohorts based on date of transplantation, as defined prior to analysis. 30-day posttransplant CDI incidence and mean LOS after transplantation were tabulated for each group. Nonparametric testing was used to compare CDI incidence and LOS between the baseline period and PDSA#2 using the χ2 and Wilcoxon rank sum tests, respectively, at a prespecified two-sided significance thresholds of *p* = .05, using SASOnDemand for Academics.

## Results

**Figure 1.**
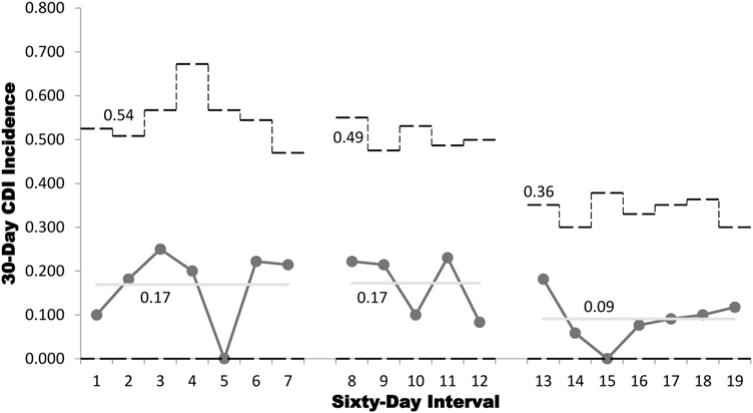
SPC p-chart analysis showing 30-day posttransplantation *Clostridioides difficile* infection (CDI) incidence with 3σ control limits, for patient cohorts sorted by transplantation date. Values reported from left to right for baseline period, plan-do-study-act cycle #1, #2.

**Figure 2.**
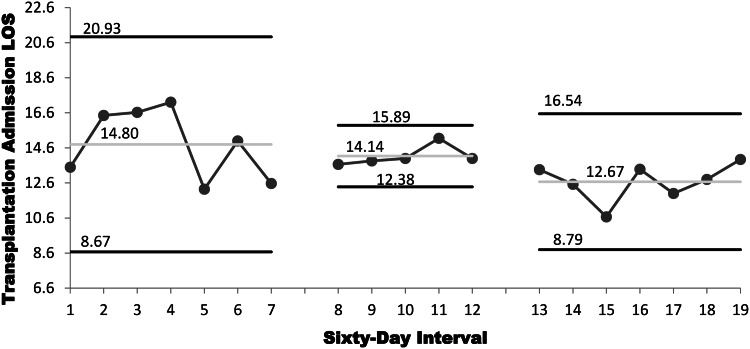
SPC x-bar analysis showing transplantation admission mean length of stay with 3σ control limits for patient cohorts sorted by transplantation date. Values reported from left to right for baseline period, plan-do-study-act cycle #1, #2.

Two-hundred eleven auto-SCTs were performed, predominantly for multiple myeloma (MM) and non-Hodgkin lymphoma (NHL). In the baseline period, 65 transplantations were conducted over 14 months (MM 39, NHL 13, other 13); in PDSA#1, 58 transplantations were conducted over 10 months (MM 29, NHL 19, other 10); and in PDSA#2, 88 transplantations were conducted over 14 months (MM 55, NHL 21, other 12). SPC p-chart analysis, as shown in Fig. 1, estimates 30-day posttransplantation CDI incidences of .17 per patient undergoing transplantation during both the baseline period and PDSA#1 and CDI incidence of .09 in PDSA#2 (Fig. 1). SPC x-bar analysis showed that mean LOS was 14.8 days at baseline, 14.1 days in PDSA#1, and 12.7 days in PDSA#2 (Fig. 2). Discrete testing did not detect a statistically significant difference in incident CDI between the baseline period and PDSA#2 at a prospectively defined threshold (χ^2^ = 2.11, *p* = .15) or LOS (*Z* = 1.79, *p* = .07).

## Discussion

In this study, we describe a quality improvement effort undertaken to reduce CDI incidence following autologous SCT at our institution. A first intervention was suspension of universal ciprofloxacin use during periods of posttransplantation neutropenia. A second was incorporation of UV tools into postdischarge room cleaning. Although other studies have concluded that commercially available UV cleaning equipment is efficacious in reducing counts of virulent bacteria on patient-room surfaces, its effectiveness in protecting immunocompromised patients is not well established.^7–9^

In our first PDSA cycle, rates of neutropenic fever increased after universal prescription of ciprofloxacin was discontinued, as previously reported.^10^ Although fluoroquinolones have been associated with CDI, several studies show that prescription in the peri-transplant period is associated with fewer episodes of febrile neutropenia and bacteremia. These effects may, in turn, reduce overall antibiotic exposure for patients receiving prophylaxis.

In a second PDSA cycle, implementation of UV cleaning tools was followed by a meaningful fall in CDI rate. Interpreting this result, which did not meet a prespecified threshold for statistical significance, is challenging. Our study relied on single-institution cohort of fixed size, precluding prospective powering and sufficient confounder control for strong causal inference. For example, differences in the diarrheagenic effects of various chemotherapeutic agents might have driven changes in CDI testing and detection over time. Numeric decreases in LOS from one PDSA cycle to the next might manifest a secular trend toward increasing outpatient management after autologous SCT. Regarding balance measures, note that institutional mortality rates after autologous stem cell transplant fell continuously throughout the study period.

Our findings contrast with those of Brite and colleagues, who did not observe even a numeric decrease in CDI rate after implementation of postdischarge UV cleaning tools. The discrepancy in our findings may reflect a true difference in treatment effect, whether as a result of differences in institutional baseline CDI rates, differences in host characteristics, or differential environmental mediators of cleaning. The discrepancy might otherwise follow from how cases were defined in each study. A continuing focus in our own institution is on separation of *Clostridioides difficile* colonization from CDI through patient factor selection and two-step testing, to better guide appropriate treatment following transplantation.

## References

[1] Alonso CD , Treadway SB , Hanna DB , et al. Epidemiology and outcomes of Clostridium difficile infections in hematopoietic stem cell transplant recipients. Clin Infect Dis 2012;54:1053–1063. 22412059 10.1093/cid/cir1035 PMC3309884

[2] Alonso CD , Marr KA . Clostridium difficile infection among hematopoietic stem cell transplant recipients: beyond colitis. Curr Opin Infect Dis 2013;26:326–331. 23806895 10.1097/QCO.0b013e3283630c4c PMC4222064

[3] Chopra T , Chandrasekar P , Salimnia H , Heilbrun LK , Smith D , Alangaden GJ . Recent epidemiology of Clostridium difficile infection during hematopoietic stem cell transplantation. Clin Transplant 2011;25:E82–E87. 20973823 10.1111/j.1399-0012.2010.01331.x PMC3860287

[4] Kamboj M , Son C , Cantu S , et al. Hospital-onset Clostridium difficile infection rates in persons with cancer or hematopoietic stem cell transplant: a C3IC network report. Infect Control Hosp Epidemiol 2012;33:1162–1165. 23041818 10.1086/668023 PMC3670420

[5] Luo Y , Zhang S , Shang H , Cui W , Wang Q , Zhu B . Prevalence of *Clostridium difficile* infection in the hematopoietic transplantation setting: update of systematic review and meta-analysis. Front Cell Infect Microbiol 2022;12:801475. 35265530 10.3389/fcimb.2022.801475 PMC8900492

[6] Guddati A , Kumar G , Ahmed S , Ali M , Kumar N , Hari P , Venu N . Incidence and outcomes of Clostridium difficile-associated disease in hematopoietic cell transplant recipients. Int J Hematol 2014;99:758–765. 24715522 10.1007/s12185-014-1577-z

[7] Liscynesky C , Hines LP , Smyer J , Hanrahan M , Orellana RC , Mangino JE . The effect of ultraviolet light on Clostridium difficile spore recovery versus bleach alone. Infect Control Hosp Epidemiol 2017;38:1116–1117. 28669367 10.1017/ice.2017.126

[8] Yang JH , Wu UI , Tai HM , Sheng WH . Effectiveness of an ultraviolet-C disinfection system for reduction of healthcare-associated pathogens. J Microbiol Immunol Infect 2019;52:487–493. 28951015 10.1016/j.jmii.2017.08.017

[9] Brite J , McMillen T , Robilotti E , et al. Effectiveness of ultraviolet disinfection in reducing hospital-acquired Clostridium difficile and vancomycin-resistant Enterococcus on a bone marrow transplant unit. Infect Control Hosp Epidemiol 2021;39:1301–1306. 10.1017/ice.2018.215 PMC8524758 30226124

[10] Grose K , Ballen KK , Kindwall-Keller TL , et al. Ciprofloxacin prophylaxis does not affect incidence of Clostridium difficile infection in autologous stem cell transplant patients. In: *Program and Abstracts of the 19th American Society of Hematology Annual Meeting*; December 1–4, 2018; San Diego, CA. Abstract 731.

